# NAD/NAMPT and mTOR Pathways in Melanoma: Drivers of Drug Resistance and Prospective Therapeutic Targets

**DOI:** 10.3390/ijms23179985

**Published:** 2022-09-01

**Authors:** Alice Indini, Irene Fiorilla, Luca Ponzone, Enzo Calautti, Valentina Audrito

**Affiliations:** 1 Division of Medical Oncology, Department of Medicine and Surgery, Ospedale di Circolo e Fondazione Macchi, ASST dei Sette Laghi, 21100 Varese, Italy; 2 Department of Science and Technological Innovation, University of Eastern Piedmont, 15121 Alessandria, Italy; 3 Department of Molecular Biotechnology and Health Sciences, Molecular Biotechnology Center “Guido Tarone”, University of Turin, 10126 Torino, Italy

**Keywords:** melanoma, drug resistance, metabolic reprogramming, NAMPT, mTOR, signaling, cancer therapy

## Abstract

Malignant melanoma represents the most fatal skin cancer due to its aggressive behavior and high metastatic potential. The introduction of BRAF/MEK inhibitors and immune-checkpoint inhibitors (ICIs) in the clinic has dramatically improved patient survival over the last decade. However, many patients either display primary (i.e., innate) or develop secondary (i.e., acquired) resistance to systemic treatments. Therapeutic resistance relies on the rewiring of multiple processes, including cancer metabolism, epigenetics, gene expression, and interactions with the tumor microenvironment that are only partially understood. Therefore, reliable biomarkers of resistance or response, capable of facilitating the choice of the best treatment option for each patient, are currently missing. Recently, activation of nicotinamide adenine dinucleotide (NAD) metabolism and, in particular, of its rate-limiting enzyme nicotinamide phosphoribosyltransferase (NAMPT) have been identified as key drivers of targeted therapy resistance and melanoma progression. Another major player in this context is the mammalian target of rapamycin (mTOR) pathway, which plays key roles in the regulation of melanoma cell anabolic functions and energy metabolism at the switch between sensitivity and resistance to targeted therapy. In this review, we summarize known resistance mechanisms to ICIs and targeted therapy, focusing on metabolic adaptation as one main mechanism of drug resistance. In particular, we highlight the roles of NAD/NAMPT and mTOR signaling axes in this context and overview data in support of their inhibition as a promising strategy to overcome treatment resistance.

## 1. Introduction

The onset of drug resistance represents a dramatic aspect in cancer management. Therefore, overcoming therapeutic resistance is one of the most pressing needs in cancer therapy [1,2,3,4]. Melanoma is an aggressive tumor originating from pigment-producing melanocytes, and is by far the deadliest form of skin cancer [5,6].

The neoplastic transformation of melanocyte is most often initiated by constitutive activation of the RAS/RAF/MAPK signaling cascade as a consequence of mutations in either BRAF (50%), NRAS (30%), or neurofibromin 1 (NF1, 10%), which collectively represent the most frequent driver mutations found in melanomas (Cancer Genome atlas network [7]).

Whereas most patients with primary melanoma are cured with surgery alone, patients with metastatic melanoma often show an initial favorable response to BRAF- and MEK-targeted therapies [8,9] and/or immune-checkpoint inhibitors (ICIs) [10,11], and eventually develop disease progression due to intrinsic or acquired resistance mechanisms [12,13,14,15]. Resistant tumors may originate from preexisting tumor subclones under selective therapeutic pressure (intrinsic/innate resistance), or from an evolutionary process taking place during treatment (adaptive/acquired resistance) [13,16,17]. Drug resistance develops through a variety of cellular mechanisms, including genetic and epigenetic alterations, aberrant activation of signaling pathways, phenotype plasticity, and metabolic rewiring [13,14,15,17,18,19,20]. Moreover, tumor heterogeneity increases the probability of developing resistant clonal cell populations under different therapeutic regimens [21]. Due to the high prevalence of resistance to BRAF and MEK inhibitors (BRAFi and MEKi) and ICIs, continued efforts toward the identification of the mechanisms at the basis of therapeutic failures remain necessary to design more efficient, durable, and personalized treatment options.

Cancer cells are endowed with the capacity to dynamically rewire their energetic metabolism to adapt to stressful conditions, such as restriction of nutrients, acidity, hypoxia, and changes in cell–cell interactions in the tumor microenvironment (TME). This elevated metabolic plasticity fosters tumor maintenance, progression, and drug resistance [18,22,23,24,25,26,27,28,29,30,31]. The activation of drivers’ oncogenic pathways in tumors grants dynamic modulation of both cytosolic and mitochondrial metabolic pathways that cause the production of elevated levels of adenosine triphosphate (ATP) during cancer progression [19,32,33].

In this review, we provide an overview of currently available therapeutic strategies for metastatic melanoma and highlight both known and potential resistance mechanisms to ICIs and targeted therapies, with particular focus on the metabolic rewiring of tumor metabolism as a major adaptation strategy that cancer cells exploit under selective drug’s pressure to escape death and that can affect also immune cell response. Specifically, we will discuss the impacts on melanoma aggressiveness and drug resistance of the de-regulation of nicotinamide adenine dinucleotide (NAD) and of its rate-limiting biosynthetic enzyme nicotinamide phosphoribosyltransferase (NAMPT) and of the mammalian target of rapamycin (mTOR) pathways, the inhibition of which holds the promise to improve the effectiveness of current first-line treatments for metastatic melanoma.

## 2. Overview of Conventional Therapies for the Treatment of Cutaneous Melanoma

Before the recent therapeutic advances, survival outcome of patients with advanced/metastatic melanoma was poor with a median overall survival (OS) of approximately 7.5 months and 5-year survival rate of ~6% [34]. Two therapeutic strategies have paved the way to improved survival in this setting [12,35]. The first one involves the modulation of the immune system with monoclonal antibodies acting as ICIs, targeting either the cytotoxic T-lymphocyte-associated antigen 4 (CTLA-4) or the programmed cell-death 1 (PD-1) [36,37,38]. Immune checkpoint molecules are key modulators of anti-tumor T cell immune response, which are present on T cells, antigen-presenting cells (APCs), and tumor cells. Their interaction activates either inhibitory or activating immune signaling pathways. The most important ICIs currently approved for the treatment of melanoma are the antibodies targeting inhibitory checkpoints on T cells, such as PD-1 (nivolumab, pembrolizumab), either alone or in combination with anti-CTLA-4 antibody (nivolumab plus ipilimumab). The second class of drugs target the mitogen-activated protein kinase (MAPK) pathway, which is constitutively active in melanomas harboring BRAF V600 mutations [9,39,40], accounting for approximately 50% of patients with cutaneous melanoma. To date, three targeted therapy combinations of BRAFi and MEKi have been approved and are routinely used in the clinic, with overall superimposable therapeutic results and slight differences in their toxicity profiles: vemurafenib and cobimetinib, dabrafenib and trametinib, and encorafenib and binimetinib [8,9,39,40].

Despite the undiscussed therapeutic gain obtained with novel drugs, the majority of patients with metastatic melanoma will still not derive durable benefit from systemic treatments. Indeed, treatment responses range from 40–60% for ICIs (alone or in combination) up to 90% with targeted therapies. However, acquired resistance eventually develops in more than half of patients after ~12 months from the beginning of treatment [41,42]. Significant efforts are presently underway in ongoing clinical trials to elucidate how to obtain the best response by combining BRAFi and MEKi, and how to sequence or combine this treatment with ICIs. One of the challenges is to identify patients who gain the largest benefit with BRAFi plus MEKi and who instead could obtain better disease control with a planned sequence or an upfront combination of ICIs in association with BRAFi plus MEKi. Ultimately, there is an intense effort to understand drug resistance mechanisms to identify novel potential targets and predictive markers. In the next sections, the main mechanisms related to the onset of resistance to ICIs and/or targeted therapy will be summarized.

## 3. Mechanisms Underlying Response and Resistance to ICIs

Overall, there is a high degree of heterogeneity in responses to ICIs. Patients can be stratified as being sensitive, or as displaying either primary or secondary resistance, the latter being furtherly subdivided in adaptive and acquired resistance [16]. Many factors explain the heterogeneity in patients’ response to ICIs [43], moreover the immune response is a dynamic process and is constantly evolving in each patient, as a result of genetic and environmental factors, and treatment interventions (i.e., surgery, radiotherapy, chemo- and immunotherapy).

### 3.1. Tumor Antigens and Mutations: The Role of Tumor Mutational Burden

Primary resistance to ICIs is common and affects up to 60% of patients among different cancer types [44]. One of the principal reasons is the lack of expression of antigens by tumor cells. The neoantigen (neo-Ag) load of a tumor can be predicted by its tumor mutational burden (TMB), with a higher TMB more likely to increase the neo-Ag load [45]. TMB is not a static variable, rather it undergoes constant dynamic changes during cancer development: loss of neo-Ag through genetic and epigenetic processes of silencing and elimination can be a mechanism of acquired resistance to ICIs [46,47]. Therefore, TMB might play a role in either primary or secondary resistance mechanisms.

### 3.2. Antigen Presentation

Alterations in mechanisms of T cell recognition for immune activation can be responsible for ICIs resistance. The loss of functional β-2 microglobulin (B2M) expression, which stabilizes the major histocompatibility complex (MHC) class I proteins on the cell surface, leading to decreased tumor cells recognition by CD8+ T cells, was first observed in patients developing resistance to adoptive T cell therapy [48]. Alterations of B2M also include mutations and loss of heterozygosity, leading to both innate and acquired resistance to ICIs [42]. Deficiencies in antigen presentation, due to alterations in HLA genes mutations, altered expression, and/or loss of diversity of HLA alleles, play a major role in resistance to ICIs [49,50,51,52]. Clinical evidence from melanoma patients receiving ICIs showed loss of MHC class I protein expression on tumor cells to be a mechanism of primary resistance to anti-CTLA4, while MHC class II protein expression predicted response to anti-PD1 therapy [53]. Immuno-editing during tumor development can drive the emergence of tumor clones with reduced MHC class I expression or production of neo-Ag with impaired capacity of binding to MHC class I molecules [54].

Variations in HLA molecules expression can partly be driven by alteration of signals through the interferon (IFN)-γ receptor. The interferon insensitivity in tumor cells can be obtained through mechanisms of epigenetic signaling of IFN-signaling components and/or increased expression of negative regulators [55]. Alterations include copy-number loss of IFN-γ receptor 1 (IFNGR1) and IFGNR2, and amplification of IFN-γ pathway inhibitors, such as suppressor cytokine signaling 1 (SOCS1) and protein inhibitor of activated STAT4 (PIAS4) [56]. Inactivating Janus kinase 1 and 2 (JAK1 and JAK2) mutations, affecting the JAK-STAT signaling pathway, can induce expression of PD-L1 ligands, reducing antigen presentation and promoting the escape from the anti-proliferative effect of IFN [57].

### 3.3. Evolution of Immune Response: T Cell Exhaustion

Adaptive resistance mechanisms during treatment with ICIs include the compensatory up-regulation of alternative checkpoints which generates an exhausted T cell phenotype. These include the T cell immunoglobulin, mucin domain-3 protein (TIM-3), lymphocyte-activation gene 3 (LAG3), B and T lymphocyte attenuator (BTLA), T-cell immunoreceptor tyrosine-based inhibition motif domain (TIGIT), and V-domain immunoglobulin-containing suppressor of T cell activation (VISTA) [58].

Along with the increased expression of inhibitory receptors, the exhausted T cells phenotype includes epigenetic modifications, leading to transcriptional changes and limited effector functions [59,60,61]. T cells exhaustion is promoted by increased PD-L1 expression on tumor cells, after prolonged exposure to anti-PD1 antibodies, and leads to an impaired T cell function [62]. Moreover, chronic Ag exposure eventually leads to the exhaustion of precursor memory T cells, thus impairing the memory formation which is responsible for durability of response [63,64].

### 3.4. T Cell Priming and Infiltration: “Hot” and “Cold” Tumors

Together with antigen creation and presentation, the main condition for the immune system to be enhanced by ICIs is the presence of an abundant and functionally active immune infiltrate within the TME [65]. Immunologically “hot tumors” display an abundant T cell effector infiltration, as well as a genetic signature characterized by intense expression of cytolytic and IFN-γ related genes [66,67,68]. Defects in dendritic cells (DCs) migration and subsequent tumor specific T cell infiltration in the TME reduce response to anti-PD1/PD-L1 antibodies in different tumor subtypes [69,70]. However, tumors can display an extensive immune infiltrate with an overall immune-suppressive profile (myeloid derived suppressor cells (MDSCs), T regulatory (regs) cells, and T helper 2 (Th2)), thus being intrinsically resistant to ICIs [71,72,73]. A complex interplay of cytokines finally determines the immune infiltration rate and the overall balance between immune stimulation and evasion within the TME [74]. Conversely, immunologically “cold” tumors show rare or absent T cell infiltrates within the TME and usually no expression of PD-1 ligands [74]. Tumor cells can activate T cell exclusion and become immune privileged, reducing T cell trafficking to the TME through mechanisms of epigenetic silencing, and post-translational modification of chemokines [74,75]. The vascular-endothelial growth factor (VEGF) acts as an immune-suppressive cytokine, increasing the expression of inhibitory molecules promoting T cell exhaustion, and creating a selective endothelial layer which reduces cytotoxic T cell trafficking and extravasation to the TME, favoring the infiltration of T regs [76,77]. Intra- and peritumoral fibroblasts reduce T cell entry by producing a dense stromal matrix through the activation of the TGF-β pathway, which also inhibits cytotoxic T lymphocytes and up-regulates T regs [78,79].

### 3.5. Genomic Correlates of Resistance: Extrinsic and Intrinsic Mechanisms

Cancer cells can themselves display autonomous resistance to ICIs, through the activation of specific oncogenes and silencing of tumor suppressor genes, influencing immune cell infiltration, cytokines production, and angiogenesis. The activation of WNT/β-catenin signaling pathway correlates with reduced T cell and CD103+ DC infiltration into the TME [80]. Loss of the tumor suppressor gene phosphatase and tensin homolog (PTEN), inducing hyper-activation of the PI3K/AKT/mTOR pathway, results in increased VEGF and IL-8 production, which recruit MDSCs and T regs [71,81]. Activating BRAF mutations are associated with a weak or absence of T cell infiltration and activation [81]. Mutations affecting the signaling molecules within the JAK-STAT pathway can induce expression of PD-1 ligands and lack of response to anti-proliferative effect of IFN [66]. Elevated lactate dehydrogenase A (LDHA) is a well-known negative prognostic factor in melanoma. There is evidence supporting the role of lactic acid as a potent inhibitor of function and survival of T and NK cells leading to tumor immune escape [82,83]. Indeed, preclinical evidence suggests that blocking LDHA in the tumor might improve the efficacy of anti-PD-1 therapy for melanoma [84].

## 4. Mechanisms Underlying Resistance to BRAF and MEK Inhibitors

The concurrent inhibition of BRAF and MEK proteins of the MAPK pathway decreases MAPK-driven acquired resistance, leading higher rate of tumor responses, more durable responses, and improved progression-free and overall survival rates. Primary resistance to BRAFi and MEKi, accounting for about 15–20% of patients with BRAF V600E mutation, is primarily related to high intratumoral heterogeneity, or loss of some tumor suppressor genes, such as PTEN and NF1 [85]. However, most commonly patients eventually develop acquired resistance during treatment, due to selective pressure of targeted therapy on tumor cells. Several molecular alterations can be responsible for secondary resistance to BRAFi and MEKi, as recently extensively reviewed by Kozar et al. [17].

### 4.1. Re-Activation of the MAPK Pathway and/or Alternative Signaling Pathways

The main cause of acquired resistance to BRAFi is the reactivation of the MAPK pathway—occurring in 80% of BRAFi-resistant tumors [86]. Reactivation of the MAPK pathway, which was found in up to 70% of melanoma cases upon disease progression, can occur at different levels [87]. The most important molecular mechanisms include: alternative splicing or overexpression of BRAF protein (13–30% of BRAFi-resistant tumors) [88]; amplification of BRAF V600E (13–20% of patients) [88,89]; overexpression of C-RAF (RAF proto-oncogene serine/threonine-protein kinase); or COT1 kinases, activating mutations in NRAS, MAP2K1, or MAP2K2 or the loss of NF1 [86]. Another alternative pathway sustaining BRAFi resistance mechanisms is the PI3K/AKT/mTOR. Mutations in AKT1, AKT3, PIK3CA, PIK3CG, PIK3R2, or PHLPP1, as well as PTEN loss, or the overexpression of multiple receptor tyrosine kinases, including epidermal growth factor receptors (EGFRs), insulin-like growth factor 1 receptors, platelet-derived growth factor receptors α and β, or fibroblast growth factor receptor 3 have been reported in resistant melanoma tumors [86].

Another signal transduction pathway involved in primary and acquired resistance to BRAFi and MEKi is the pleiotropic hepatocyte growth factor (HGF) [90]. HGF secretion by stroma cells within the tumor and its paracrine signaling to melanoma cells, increasing intracellular signaling and RAS expression, finally activated the MAPK signaling pathway, through the downstream activation of the MAPK/ERK and PI3K/AKT transduction pathways. HGF also contributes to on-treatment resistance mechanisms by decreasing the expression of genes encoding pro-apoptotic proteins [90,91].

The CCND1 (BCL1) gene, encoding cyclin D1 is a key protein for cell cycle regulation (G1 to S phase transition), and also plays a major role in BRAFi resistance mechanisms in melanoma cells [92]. Cyclin D1 overexpression leads to BRAFi resistance and this phenomenon is enhanced when both cyclin D1 and CDK4 are overexpressed [92]. The evidence that approximately 20–38% of melanoma show CCND1 amplification, thus potentially being primarily resistant to BRAFi, has suggested that adding a CDK4/6 inhibitor could enhance the efficacy of BRAFi and MEKi [92,93].

### 4.2. Therapy-Mediated Selection of Resistant Tumor Cell Subpopulations

Epigenetic mechanisms alone, or combined with genetic alterations, which account for the intra-tumor heterogeneity, may impact on disease evolution and progression and promote acquired resistance to BRAFi and MEKi in melanoma cells [94,95]. Drug resistance can arise by a selection of pre-existing subclones possessing stem cell-like properties that are able to withstand drug treatment [96]. Alternatively, cancer cells can become resistant by acquiring genetic mutations or by rewiring the epigenome (DNA methylation and histone modifications mechanisms) or metabolome (metabolic adaptation mechanisms) under drug treatment-mediated selection pressure [95,97,98]. The observed adaptations of cancer cells are at the basis of the concepts of tumor cell plasticity and phenotypic switching meaning that melanoma cells are not only capable of rapidly adapting to therapies by acquiring mutations, but they also tend to switch their molecular and cellular phenotype in order to overcome drug treatment. The most common phenotypic changes that melanoma cells undergo to escape inhibition are linked to epithelial-to-mesenchymal transition (EMT), differentiation/de-differentiation, changes in proliferation rates, and metabolic rewiring, modulating the activity of the master melanocyte transcription factor microphthalmia-associated transcription factor (MITF) and the receptor tyrosine kinase (RTK) AXL [99,100,101], among others.

Due to this complexity, overcoming treatment resistance is challenging, and often requires targeting different pathways to obtain therapeutic results. In the following paragraphs, we provide an overview of metabolic adaptation mechanisms linked with drug resistance in melanoma, often associated with emerging alteration of nicotinamide adenine dinucleotide (NAD) metabolism and mTOR signaling.

## 5. Metabolic Rewiring in Tumors

Cancer cells possess high metabolic plasticity widely recognized as one of the hallmarks of cancer [1,4,18,19,102]. Tumors are able to dynamically adapt their metabolism to the nutrient restriction and changes, acidity, hypoxic conditions, and cell–cell interactions within the TME [31,103,104,105]. At the same time, the activation of intrinsic oncogenic pathways in tumor drives dynamic modulations of both cytosolic and mitochondrial metabolic pathways in order to produce elevated levels of ATP during cancer progression [19,32,33,106].

The first who described the phenomenon of metabolic adaptation was Otto Warburg in 1920, which observed a metabolic switch in cancer cells, known as “Warburg effect”. According to this model, cancer cells do not take advantage of the mitochondrial oxidative phosphorylation (OXPHOS) to produce energy, but showed high rates of glucose uptake and lactate secretion, even in the presence of oxygen (i.e., aerobic glycolysis) [25,107,108]. Specifically, pyruvate is catabolized into lactate by LDHA. LDHA is associated with cancer progression and poor prognosis, since its overexpression promotes angiogenesis and tumor dissemination [109,110]. In neoplastic diseases, the high activity of LDHA produces large amounts of lactate, which is secreted by cancer cells. Secreted lactate is necessary for supporting angiogenesis, immune escape, cell migration, metastasis, and metabolic self-sufficiency of cancer cells [111,112].

Recently, it became clear that aerobic glycolysis/Warburg effect is not the only metabolic pathway used by cancer cells to sustain their growth. Rather, mitochondrial metabolism emerged as a crucial player in promoting malignant transformation [113,114]. Mitochondria are the “powerhouse of the cell” as they produce most of the ATP needed to sustain cellular processes, but also function as stress sensors and are responsible for the adaptation of the cell to environmental conditions [115]. Despite their physiological roles, mitochondria can also contribute to tumorigenesis and cancer progression producing high levels of reactive oxygen species (ROS) and metabolites driving the accumulation of DNA mutations and the activation of oncogenic signaling pathways [114,115,116]. In addition, the ability of some cells to limit mitochondrial outer membrane permeabilization (MOMP) causes mutagenesis and oncogenic transformation [117,118]. Another important player is the transcriptional coactivator peroxisome proliferator-activated receptor gamma coactivator-1 alpha (PGC-1α), the master regulator of mitochondrial metabolism, which supports mitochondrial biogenesis and OXPHOS flux, thus allowing tumor cells to produce large amounts of energy needed for growth [119,120].

However, the metabolic flexibility of cancer cells is much more complex and comprises several other pathways. Cancer cells compensate for glucose deprivation by increasing protein and amino acids scavenging, to ensure the continuous supply of nutrients [102,104,106,121]. For example, glutamine (synthesized de novo or imported from the extracellular environment) is the major amino acid required by cancer cells for proliferation [102,122,123]. Fatty acids (FAs) are another fundamental source of energy for cancer cells [124,125]. For example, it has been demonstrated an increased reliance of cancer cells on de novo biosynthesis and exogenous FA uptake to not only sustain their rapid proliferative rate, but also provide an essential energy source during conditions of metabolic stress [126]. Lastly, acetate, efficiently converted by acetyl-CoA synthetases into acetyl-CoA, is considered a regulator of cancer cell stress [127]. Acetate can be used by cancer cells to fuel tumor respiration, supporting energy and biomass production [128,129]. This metabolic dependence of cancer cells on alternative nutrients to support energy production may offer opportunities for the development of novel targeted therapies to hit multiple tumor vulnerabilities [130].

## 6. Metabolic Plasticity in Melanoma Drives Resistance

**Figure 1:**
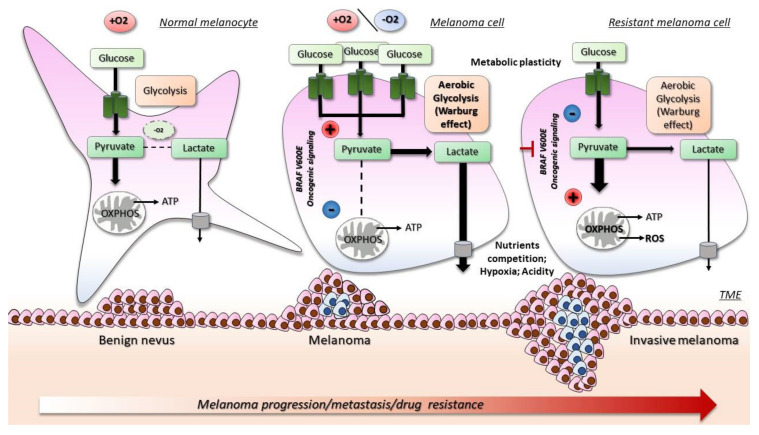
Metabolic plasticity in melanoma. In normal melanocyte, the energetic metabolism, in the presence of oxygen (O2), starts from the consumption of glucose via glycolysis to the final production of adenosine triphosphate (ATP) mainly via oxidative phosphorylation (OXPHOS). During the development of the tumor, the metabolic pathways drastically change. In melanoma cells, the BRAF-mutated oncogenic pathway, leading to the over-activation of MAPK, sustains mainly aerobic glycolysis/Warburg phenotype with secretion of lactate. Inhibition of MAPK pathway decreases glycolysis, leading to a dependence on mitochondrial metabolism with increased production of reactive oxygen species (ROS). This phenotype switch is a metabolic adaptation mechanism developed by cells to survive under drug’s pressure and is a common feature of melanoma cells resistant to targeted-therapy. Intrinsic (mutations, intracellular pathways) and extrinsic factors (nutrients, O2 levels, acidity, and soluble factors) can contribute to increase the metabolic plasticity of melanoma cells selecting subclones with a different metabolic phenotype. The progressive metabolic reprogramming in melanoma is accompanied by a drastic increase in tumor aggressiveness. +: positive regulation; −: negative regulation.

Melanoma cells, in the early stages of development, metabolize up to 80% of glucose into lactate (showing classical Warburg phenotype), and the following hypoxic conditions potentiate this metabolic process [24,131,132,133,134,135]. Lactate and protons secretion due to the overactivation of glycolysis drastically alters the melanoma microenvironment, facilitating angiogenesis, promoting melanoma metastasis, and suppressing the immune system [136,137]. It is clear that BRAF oncogenic signaling has emerged as a critical regulator of this metabolic pathway in melanoma (Figure 1). BRAF mutations lead to MAPK pathway hyperactivation and subsequent stimulation of transcription factors, such as MYC, hypoxia inducible factor-1α (HIF-1α) also induced by PI3K/AKT/mTOR, and Wnt/β-catenin signaling [22,131], which are key regulators of glycolysis, inducing transcription of several genes involved in glucose metabolism (i.e., glucose transporter 1 (GLUT1), hexokinase (HK2), LDH) [30,132,138]. In parallel, BRAF mutations can block OXPHOS, repressing expression of MITF and its target PGC-1α [27,139,140]. A second mechanism suppressing OXPHOS is due to pyruvate dehydrogenase kinase (PDK) and pyruvate dehydrogenase (PDH) axis [141]. PDK, a target gene of HIF-1α, controls and inhibits the PDH activity in the oxidation of glucose-derived pyruvate, lowering mitochondrial functions [142,143]. However, it is noteworthy that melanoma is a highly heterogeneous tumor, and subsets of melanoma possess an oxidative metabolism, correlating with poorer survival, progression, and metastasis [23,24,27,28]. Higher OXPHOS in subgroups of melanoma is driven by elevated expression of PGC-1α and improved ability to survive under conditions of oxidative stress [28]. It is, therefore, clear that both glycolysis and OXPHOS play a significant role in metabolic rewiring of melanoma cells, and that there is a dynamic switch and plasticity between these two metabolic phenotypes, according to intrinsic factors and TME conditions [23,28,113,134,144,145].

Acute treatment with BRAFi and MEKi rapidly and significantly reduces glucose uptake and inhibits the glycolytic pathway, both in vitro and in vivo [30,132,143]. Melanoma cells attempt to survive under drug’s pressure switching from glycolysis to a mitochondrial metabolism (Figure 1) [26,135,138]. In general, this is obtained through enhancement of mitochondrial activity and mitochondrial content reactivating the MITF/PCG-1α axis [26,27,28,139,140]. One hypothesis to explain this phenomenon is that this metabolic oxidative profile pre-exists in some clones prior to treatment with BRAFi/MEKi and that treatment simply selects them. The alternative possibility is that resistance is acquired as a consequence of treatment, following drug exposure.

This adaptive metabolic program limits the efficacy of targeted-therapy and is one of the mechanisms responsible for adaptive resistance [26,27,28,29,30,146]. As a consequence of high mitochondrial activity, resistant cells show higher level of ROS, which can lead to a proliferative advantage [147,148]. In addition, amino acids biosynthetic pathways, including serine, glycine, and glutamine, may contribute to melanoma metabolic rewiring in melanoma [135,146,149], as well as the metabolic exchange within TME between melanoma and stromal/immune cells [31], even if less studied during the onset of resistance.

Overall, it is clear that metabolic plasticity confers to melanoma cells a significant advantage to adapt their growth to different environmental conditions and to increase their survival even under drug-imposed selective pressures.

## 7. NAD/NAMPT Axis in Cancer

Appropriate NAD level is a critical factor to maintain efficient redox-reactions essential to generate adequate energy (i.e., ATP molecules), as well as to sustain NAD-consuming enzyme activities (i.e., mono and poly-ADP-ribose polymerases (ARTs, PARPs), CD38/CD157 and sirtuins), orchestrating fundamental biological processes, including DNA repair, apoptosis, calcium signaling, gene expression, transcription, immune regulation, circadian rhythm, and cell cycle progression [150,151]. In tumors, many of these processes are de-regulated, for these reasons, a fine-tune regulation of NAD levels is a critical element during malignant transformation and progression [150,152]. Additionally, NAD metabolism is fundamental in modulating the activation/response of immune cells, including T cells, TAMs, and MDSCs, within the TME, as recently reviewed [153,154,155,156]. NAD homeostasis is the result of a dynamic balance between its biosynthesis and consumption [157,158]. Cells maintain adequate NAD levels through multiple biosynthetic enzymatic pathways; however, a common strategy of several tumors is to sustain NAD production over-expressing its rate-limiting and vital enzyme NAMPT that converts the NAD precursor nicotinamide in nicotinamide mononucleotide (NMN), as first step in NAD regeneration [150]. Therefore, NAMPT is a critical factor involved in the modulation of cellular metabolism but also signaling pathways, as extensively reviewed in [155,159,160,161].

NAMPT exists also as extracellular form (eNAMPT) and can act as growth factor, cytokine and adipokine, regulating inflammation [152,159,161]. Emerging evidence fixes eNAMPT as a novel damage-associated molecular pattern protein (DAMP) with a remarkable role in the inflammatory response and cancer promotion [155,156,160]. It has been recently reported that eNAMPT could directly binds to membrane receptors, with C–C chemokine receptor type 5 (CCR5) and Toll-like receptor 4 (TLR4) acting as receptor antagonist and agonist, respectively, and modulating specific intracellular signaling cascades [162,163,164,165,166,167,168]. Even if we cannot exclude eNAMPT enzymatic activity, probably the most accredited hypothesis demonstrated by several studies in different cellular models was that eNAMPT could exerts cytokine-like functions independently from its NAD-biosynthetic activity, as reviewed in [160].

iNAMPT over-expression, as well as increased circulating levels of eNAMPT, were documented in several solid tumors and hematological malignancies, as reviewed in [30,156,169,170]. Several transcriptional and post-transcriptional mechanisms regulate NAMPT expression and activity in tumors, as well as multiple signals, including hypoxia, stress conditions, DNA damage, and pro-inflammatory cytokines, can induce its release [160]. In addition, rather than being mutated, the NAMPT gene was found amplified in cancer [169,171].

Functionally, NAMPT, as an intracellular and extracellular factor, impacts on tumor cell biology increasing tumor aggressiveness, correlating with worse prognosis and regulating different processes including metabolic adaptation, DNA repair, gene expression, signaling pathways, cell growth, invasion, stemness, EMT program, metastasis, and angiogenesis [154,155,156,159,170,172,173].

An emerging function of NAMPT is related to an enhanced acquired resistance to chemotherapeutic agents, and more recently to targeted therapy. NAMPT overexpression confers resistance to specific drugs (chemotherapeutic agents and selective kinase inhibitors) in several tumor models, including glioma [174], colon cancer [175], sarcoma [176], breast cancer [177,178], and melanoma [160,179], as detailed in the next paragraph. On the contrary, its inhibition increases the susceptibility to different drugs, as described in prostate cancer [180], breast cancer [181], glioma [182], leukemia, and myeloma [183,184,185]. All these evidence support the targeting of NAMPT as a novel therapeutic strategy to enhance the efficacy of chemotherapeutic agents and targeted-therapy in tumors, as well as its potential diagnostic significance in cancer and its association with a poor survival rate.

Recently, different studies have linked NAMPT/NAD axis to immune activation and regulation [153,154,155,156]. NAMPT expression can be regulated by different inflammatory signals in several types of tumor and immune cells, and, in turn, eNAMPT increases the levels and the release of pro-inflammatory cytokines IL6, IL10, IL1β, and tumor-necrosis factor-α (TNF-α) from leucocytes [153,154,155]. NAMPT-dependent NAD generation regulates the metabolic adaptation of myeloid cells and T lymphocytes. In inflammatory M1 macrophages, high levels of cytosolic NAD seem to be necessary for the efficient activation of myeloid function, while in M2-like macrophages and TAMs NAD decreases [186], as well as in exhausted tumor-infiltrating lymphocytes (TILs) [187]. Recent papers have demonstrated the feasibility of using nutrient supplementation, including NAD precursors, to improve immune cell function in multiple disease models, including cancer [187,188,189]. The manipulation of NAD levels, using NAMPT inhibitors (NAMPTi), as described in detail in the next section, can therefore impact also on immune response and this is an important point to be taking in account thinking to the potential use of NAMPT inhibition strategy in cancer therapy, maybe combining NAMPTi to target tumor cells more sensitive with NAD/NAM supplementation to preserve/enhance T cell functions. The better pharmacological approaches will need to be defined in pre-clinical studies, in selected tumor models.

### 7.1. NAMPT Inhibition in Cancer Treatment

In recent years, many specific NAMPTi have been developed mainly for oncologic diseases, and some of them are currently in clinical trials (www.clinicaltrials.gov; [151,154,155,170,190]). NAMPT inhibition leads to the reduction in NAD content in the cell, resulting in metabolic dysfunction and inhibition of ATP synthesis. The biological consequence is a block of tumor cell proliferation and cell death, mainly by apoptosis [151,154,155,156,190]. The first chemical compound developed as NAMPTi was FK866/APO866, which demonstrated important cytotoxicity and tumor regression activity both in vitro and in vivo tumor models [190,191,192,193]. A second specific NAMPTi studied was GMX1778/CHS-828 (known as prodrug GMX1777), which highlighted beneficial effects in breast and lung cancer in vitro and in vivo [193,194,195]. Despite these important results obtained with these compounds, phase I clinical trials in advanced solid tumors and leukemia showed no objective tumor remission and toxic effects, mainly thrombocytopenia and gastrointestinal symptoms [151,155,160,190]. One possible explanation of the partial failure of NAMPTi treatment could be linked to the concomitant expression of others NAD-biosynthetic enzymes (i.e., nicotin acid prosphoribosyltransferase (NAPRT)) that can overcome NAMPT inhibition ensuring NAD production from nicotinic acid and restricting the use of NAMPTi as single agents [151,160,190,193].

Next generation NAMPTi, including GNE617 and its analogues GNE618, GNE643, and GNE875 [196]; MPC5319528 and its analogue MPI0479883 and STF-118804 [197]; despite their powerful antitumor effect have not entered clinical trials because of toxicity and solubility problems due to their lipophilic chemical structures [190,198]. The strategy to overcome these limits was to improve chemical structure to obtain NAMPTi less toxic and more soluble, such as GPP78, MV78, and MS1-31 [199,200,201,202], even if it is clear that it is necessary to select tumor with low NAMPT expression to avoid compensation mechanisms. The last and novel NAMPTi developed and entered in clinical trials were: (i) the dual inhibitor of NAMPT and of the serine/threonine p21-activated kinase 4 (PAK4) named KPT-9274 that has been proposed as a candidate drug in tumors that express high levels of PAK4 and NAMPT [155,190,203,204]. This NAMPTi entered phase 1 clinical trials for patients with advanced solid tumors or Hodgkin’s lymphoma in combination with niacin and nivolumab (ani-PD-1) and as monotherapy (clinicaltrials.gov; NCT02702492, NCT04281420, NCT04914845). (ii) OT-82 is another new NAMPTi that entered phase 1 clinical trials in 2019 to assess safety and efficacy in patients with relapsed or refractory lymphoma (clinicaltrials.gov; NCT03921879) [205]. In preclinical trials, OT-82 demonstrated stronger activity towards hematopoietic malignancies compared to solid tumors [155,190,205]. OT-82 enhances the efficacy of other drugs, such as cytarabine and dasatinib. Studies in mice and nonhuman primates indicated that OT-82 was well tolerated and showed no cardiac, neurological, or retinal toxicity [205,206,207].

### 7.2. NAMPT Over-Expression in BRAFi Resistant Melanoma and Its Targeting

**Figure 2:**
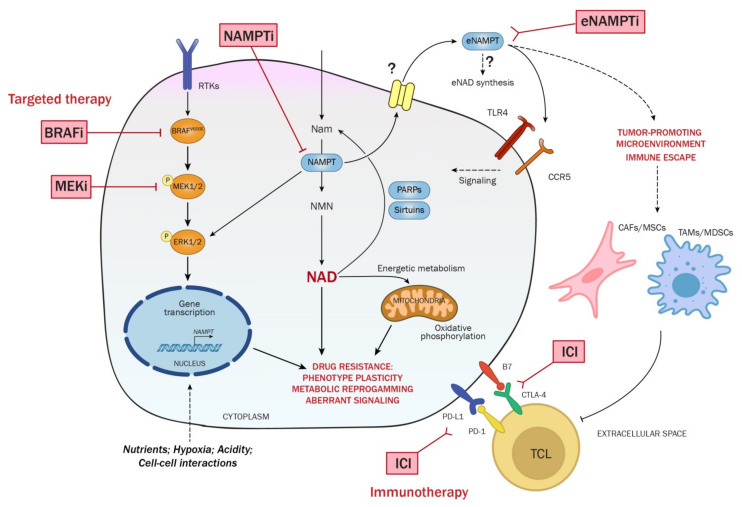
Summary of the cellular processes regulated by NAD/NAMPT axis and its connections with the BRAF/MEK/ERK signaling cascade. NAMPT is the rate-limiting enzyme in NAD biosynthesis starting from nicotinamide (Nam) that, in turn, is released by NAD-consuming enzymes (PARPs and Sirtuins mainly involved in DNA repair and epigenetics). The BRAF oncogenic signaling and NAMPT/NAD pathway are connected in melanoma: the activation of BRAF-mutated pathway promotes NAMPT transcription and NAD metabolism; on the other hand, NAMPT overexpression support MAPK activation in a positive loop. Increased levels of NAMPT and NAD sustains energetic metabolism and drives drug resistance mechanisms. NAMPT can be also released by melanoma cells, through unknown mechanisms, acting as cytokine binding to TLR4 and/or CCR5 and triggering intracellular signaling. In the microenvironment of melanoma the function of eNAMPT has not been well established, however, in other tumor models it can create immunosuppressive and tumor-promoting conditions modulating immune responses. Note that NAMPT inhibition (using pharmacological agents and/or neutralizing antibody) can play a role in blocking melanoma growth and progression, counteracting BRAF inhibitors resistance and impacting on tumor microenvironment. i: inhibitors; ICI: immune checkpoint inhibitors; NMN: nicotinamide mononucleotide; TLR4: Toll-like receptor 4; CCR5: C–C chemokine receptor type 5; TCL: T cytotoxic lymphocyte; CAFs: cancer-associated fibroblasts; MSCs: mesenchymal stem cell; TAMs: tumor-associated macrophages; MDSCs: myeloid-derived suppressor cells.

NAMPT over-expression as intracellular enzyme in melanoma compared to melanocytes has been demonstrated by several studies [179,208,209,210]. Transcriptionally, NAMPT is regulated by the activation of the BRAF oncogenic signaling, and, in turn, NAMPT overexpression can amplify and sustain MAPK and downstream pathways activation [211,212,213]. Recently, Audrito et al. showed that patients with BRAF-mutated melanoma have increased NAMPT mRNA and protein levels more than the BRAF-wild type ones [171], indicating a direct molecular link between a driver oncogenic signaling and the regulation of the expression of this enzyme. Functionally, NAMPT over-expression promotes signaling activation, proliferation and colony-formation capacity, phenotypic plasticity supporting mesenchymal/invasive transition and stem cell-like features, and supports tumorigenicity in vivo [212] (Figure 2). NAMPT over-expression induces also metabolic rewiring financing increased NAD levels, transcriptomic, and epigenetic reshuffling that steer melanoma cells toward an invasive phenotype associated with the onset of resistance to targeted therapies [179,212,214]. In BRAF-mutated melanoma cells, the inhibition of NAMPT, using FK866 and GMX1778, or its genetic knock-down, reduced cancer cell proliferation, both in vitro and in vivo [179,212,214], highlighting NAMPT as an actionable target for melanoma patients with BRAF mutations [171] (Figure 2).

### 7.3. eNAMPT as Biomarker and Functional Soluble Factor in Melanoma

Grolla et al. first showed that melanoma cell lines, or melanoma xenografts, actively secrete eNAMPT, with autocrine effects including activation of MAPK, AKT, NF-kB pathways, and increase in colony-formation in anchorage-independent conditions [211] (Figure 2). Three years later, eNAMPT was measured in patient plasma where it correlated with disease burden, response to targeted-therapy and overall survival [215]. NAMPT/NAD axis has an impact on modulating immune response [153]. Paracrine effects of eNAMPT on stromal and immune cells were described in other tumor models. For example, eNAMPT is able to polarize macrophages in M2-like type in chronic lymphocytic leukemia [216,217], and forces the mobilization of immature MDSC and enhances their production of suppressive nitric oxide in fibrosarcoma and breast carcinoma mouse models [218]. The functional role of eNAMPT within the melanoma microenvironment was only partially studied. Pylaeva et al., in 2019, highlighted a function of NAMPT as a critical molecule in priming pro-tumor functions of tumor-associated neutrophils (TANs), including tumorigenic conversion of TANs and their pro-angiogenic switch [219]. The evaluation and the correlation of eNAMPT levels and tumor infiltrating immune cells in melanoma tissue and in plasma from patients will be very interesting to clarify the impact of NAMPT on immune response in basal condition and after immunotherapy.

Intriguingly, very recently in liver carcinoma NAMPT/NAD axis was found to impinge on the IFN-γ/STAT1 axis, via NAD-dependent epigenetic mechanism potentiating IFN-induced PD-L1 expression and immune evasion [220,221]. In addition, Colombo et al. published this year that eNAMPT selectively boosts IFNγ-driven macrophage M1 polarization via transcriptional activation of STAT1/3. Importantly, the secretion of eNAMPT promotes the chemotactic recruitment of myeloid cells, therefore providing a potential positive feedback loop to foster inflammation. Last, they report that these events are independent of the activation of TLR4, at least in the cellular model of peritoneal macrophages (PECs) from mice and normal human monocyte-derived macrophages [222]. In a melanoma context, it is known that resistance to targeted therapy and ICIs may depend on common and/or selective underlying mechanisms. For instance, BRAFi/MEKi resistance is associated with changes in the tumor immune landscape that result in cross-resistance to anti-PD-1/PD-L1 immunotherapy [223]. Moreover, subsets of BRAFV600E metastatic melanoma cells that display a “native immune gene signature” suggestive of IFN pathway activation, have enhanced BRAFi resistance and sensitivity to inhibition of protein kinases involved in IFN induction [224]. Therefore, the impact of eNAMPT signaling in triggering IFN-like responses in melanoma merits to be evaluated to investigate a potential NAMPT-dependent T cell and/or macrophage function modulation and response to immunotherapy, as well as the potential role of eNAMPT as prognostic and predictive biomarker of therapeutic response to ICIs. Of note, pre-clinical evaluation of neutralizing eNAMPT antibodies could be useful to address the eNAMPT functions [155], as demonstrated in other disease models [225,226,227,228,229]. Moreover, more polar NAMPTi blocking eNAMPT enzymatic activity [199], as well as recombinant NAMPT mutants enzymatic deficient or unable to bind TLR4 [230] could be exploited to shed light on eNAMPT functions.

Overall, these data confirm that NAMPT plays a central role in phenotypic plasticity of melanoma and during the onset of resistance, suggesting a rational to target this enzyme in melanoma patients in combination with targeted- and immuno-therapy (Figure 2).

## 8. mTOR Complexes and Their Regulation

mTOR is a serine/threonine protein kinase belonging to the phosphatidylinositol 3-kinase-related kinases (PIKK) family and nucleates at least two multi-protein complexes, namely mTOR complex-1 (mTORC1) and mTOR complex-2 (mTORC2). The two mTOR-containing complexes have distinct roles and regulate a plethora of biological processes, such as cell growth, proliferation, protein and lipid synthesis, cancer, aging, lysosome biogenesis, immune modulation, and stress response.

mTORC1 is composed of five protein subunits (including mTOR): two subunits shared with mTORC2, namely mLST8 (mammalian lethal with Sec13 protein 8) [231] and DEPTOR (DEP-domain-containing mTOR-interacting protein) [232], and two exclusive subunits, Raptor (regulatory-associated protein of mTOR) [233] and PRAS40 (proline-rich Akt substrate 40 kDa) [234]. The presence of growth factors and nutrients (such as amino acids) promotes mTORC1-dependent protein synthesis through phosphorylation of two key protein substrates: 4E-BP1, which then dissociates from eIF4E, leading to initiation of protein translation, and S6K1 protein kinase.

Upstream signaling by amino acids leads to localization of mTORC1 at the lysosomal surface, where it becomes activated by GTP-bound Rheb [235]. The amount of GTP-bound, active Rheb is negatively regulated by the GAP activity of tuberous sclerosis-1 and -2 (TSC1/TSC2) protein complexes that stimulate the intrinsic GTPase activity of the protein. Phosphorylation by Akt and ERKs of TSC complexes leads to their inactivation and, thus, promotes the accumulation of the GTP-bound form of Rheb with subsequent activation of mTORC1 at the lysosomal surface. Both growth factors and nutrients converge on the activation of mTORC1. Therefore, starvation acts as a physiological inhibitor of this signaling complex. mTORC1 suppresses ULK1 (Unc-51-like kinase)-dependent autophagy, and, therefore, physiological mTORC1 inhibition potently activates autophagy [236,237]. mTORC1 also regulates de novo lipid biosynthesis and lysosomal biogenesis [238].

Sustained mTORC1 activation induces negative feedback mechanisms that desensitize cells to growth-factors-dependent stimuli. Best characterized example is the mTORC1-dependent degradation of the IRS1 adaptor protein, a protein that couples PI3K activation to insulin/IGF (IIS) signaling. Following IIS stimulation, IRS1 is targeted to proteasomal degradation by phosphorylation by S6K1 [239], thus attenuating IIS signaling. Moreover, additional mTORC1-dependent mechanisms of growth factor signaling inhibition have also been described [239]. These negative feedback mechanisms limit, in time, the cellular responses to growth factors, and their abrogation via mTORC1 inhibition favors rebound activation of the PI3K to Akt signaling and stimulation of cell survival [240].

mTORC1 is also inhibited by different forms of cellular stress. Prolonged mTORC1 signaling activation leads to the erosion of cellular energy stores due to consumption of ATP during anabolic processes. Therefore, intrinsic to the response of cells to stress is the limitation of energy expenditures to favor survival versus growth via attenuation of mTORC1 signaling activity [241]. In particular, a drop in cellular ATP content leads to activation of the LKB1/AMPK axis that ultimately inhibits mTORC1 signaling via multiple mechanisms (reviewed in [242]). Genotoxic stress also leads to a p53-dependent mTORC1 inhibition [243,244]. Additionally, mTORC1 is also inhibited under hypoxic conditions [245,246].

mTORC2 complex is composed of mTOR, mLST8, DEPTOR, Sin1/MAPKAP1 (mammalian stress-activated MAPK-interacting protein 1), Rictor (rapamycin-insensitive companion of mTOR), and Protor1/2 (protein observed with Rictor 1/2) [239]. The best established function of this complex is the phosphorylation of AGC kinases, such as Akt/PKB, SGK1 (serum/glucocorticoid-regulated kinase 1), and PKCα (and possibly other PKC isoforms). mTORC2 phosphorylates Akt at Serine 473, located in the hydrophobic regulatory motif, which leads to full Akt kinase activation. Akt regulates cell survival, proliferation, and energetic metabolism via multiple protein substrates, such as TSC2, GSK3β (glycogen synthase kinase 3β), and FoxO proteins. SGK1 regulates ion transport and apoptosis [247], while PKCα controls cytoskeleton organization and cell motility [248]. Like mTORC1, mTORC2 is also involved in the regulation of cell metabolism in several tissues by promoting glycolysis, lipid synthesis, and amino acid transport [239]. mTORC2 regulation is primarily achieved by growth factor signaling through the PI3K pathway. The link between growth factor signaling, PI3K, and mTORC2 is provided by its integral component, Sin1, which possesses a pleckstrin homology domain that inhibits mTORC2 in the absence of active PI3K signaling. Binding to PI3K lipid products phosphatidyl inositol (3,4,5) triphosphate (PIP3) at the plasma membrane releases this inhibition and fosters interaction between mTORC2 and its Akt protein substrate, which is also recruited at PIP3-enriched membrane domains. Cross-phosphorylation between Akt and mTORC2 further modulates their activation and subcellular distribution [249,250]. Interestingly, distinct subcellular pools of mTORC2 at the plasma membrane, mitochondria, and endosomal vesicles have been described, which possess a differential dependence on growth factor signaling and PI3K activity (reviewed in [251]).

### 8.1. mTOR Inhibition in Cancer Treatment

mTOR signaling is frequently hyperactivated in the majority of human solid tumors as a consequence of mutations in either the PI3K/Akt or the RAS/MAPK signaling modules (for review see [252]).

A plethora of preclinical data has indicated mTOR inhibition as a rational approach in oncology. The most extensively used mTOR inhibitor is Rapamycin, a macrolide that causes dissociation between mTOR and RAPTOR proteins within mTORC1 complex [239] and which has long been considered as an mTORC1-selective inhibitor. It is now well established, however, that prolonged rapamycin treatment also inhibits mTORC2 due to the gradual sequestration of newly formed mTOR moieties [240]. Rapamycin and its “Rapalogs” derivatives, however, only partially block the activity of mTORC1, having a limited effect on 4EBP1 phosphorylation, an essential event for CAP-dependent mRNA translation at the ribosome [253]. Rapamycin and Rapalogs derivatives (sirolimus, everolimus and temsirolimus) are the only mTOR inhibitors currently approved for clinical usage [254].

A second class of mTOR inhibitors, including Torin1 and PP242, also known as Torkinib, has been developed. These compounds function as ATP-competitive inhibitors of the mTOR protein kinase, blocking both mTORC1 and mTORC2 downstream signaling, thus displaying a broader range of action than rapamycin [255,256]. Within this class, AZD2014, INK-128, and AZD8055 are currently under scrutiny in clinical trials for several types of tumors. Dual PI3K/mTOR inhibitors, such as NVP-BEZ235, PQR309, and PK1587, were also entered in clinical trials [257].

Third-generation mTOR inhibitors (such as Rapalink 1) currently under preclinical development, combine the durable effect of rapamycin and dual mTORC1/mTORC2 inhibition. Notably, this class of compounds show preclinical efficacy even on cancer cells bearing constitutively active mTOR mutations that cause resistance to mTOR kinase inhibitors [258].

Treatment with mTOR pharmacological inhibitors as stand-alone therapy failed to provide long-lasting benefits in the majority of cancer patients (for review see [259]), including those suffering from melanoma [260,261]. The reasons for the poor efficacy of mTOR inhibition in oncotherapies rely on both cell-autonomous and cell-nonautonomous mechanisms [262]. Firstly, all available mTOR inhibitors have mostly a cytostatic but not cytotoxic effect on tumor cells. One major adaptive mechanism that grants to tumor cells the capacity to survive mTOR inhibition by Rapalogs consists of the activation of the pro-survival RTK/PI3K/Akt signaling axis, due to interruption of the negative feedback emanating from mTORC1 (reviewed by [242]). Akt reactivation triggered by mTOR inhibition occurs also with mTOR kinase inhibitors. Yoon et al. found that in BRAF-mutated melanoma cells treated with Torin-1, Akt regains phosphorylation at its regulatory sites, despite a complete suppression of mTORC2 activity [263]. This is achieved through Integrin-linked kinase (ILK), which, together with other protein kinases, can vicariate for mTORC2 deficiency and promote “paradoxical” Akt phosphorylation at Ser473 [264,265,266]. Additionally, tumor cells can be protected by various forms of stress via autophagy, which can be triggered by mTOR inhibition. Although autophagy can play tumor-suppressive functions, it is believed to exert mostly pro-tumorigenic functions in advanced melanoma [267,268].

mTOR inhibition can also enhance the maintenance and self-renewal of various stem cell types (reviewed in [269]), and the relapse of advanced melanomas is believed to rely, at least in part, on “dormant” de-differentiated cancer cells with stem cell-like features. Dormant melanoma cells are characterized by low mTOR activity, and, in turn, mTOR inhibition is sufficient to promote cell cycle pausing and dormancy (see [270]).

The presence of mutations that render mTOR kinase refractory to inhibition by Rapalogs or mTOR kinase inhibitors is another potential mechanism of the resistance of melanoma cells to mTOR inhibition, although this represents a relatively rare event [271].

Among cell-non autonomous mechanisms that limit the efficacy of mTOR inhibitors in cancer therapy are potential immunosuppressive effects. Rapamycin, the prototypical mTOR inhibitor, is used to prevent organ rejection in transplanted patients [254,272]. mTOR signaling plays important functions in the activation of native immunity, since mTORC1 and mTORC2 mediate Type I and -II IFN-biological responses in normal and malignant cells (for review see [273,274]). However, mTOR signaling plays complex and cell type-specific roles in adaptive immunity, as depending on the effector cell type, it may have immunostimulatory or immunosuppressive functions. The complexity of mTOR signaling in shaping anti-tumor immune responses has been extensively reviewed elsewhere [254,275,276], and is beyond the scope of the present review.

### 8.2. mTOR Signaling and the Breakdown of the Senescence Barrier in Melanomagenesis

**Figure 3:**
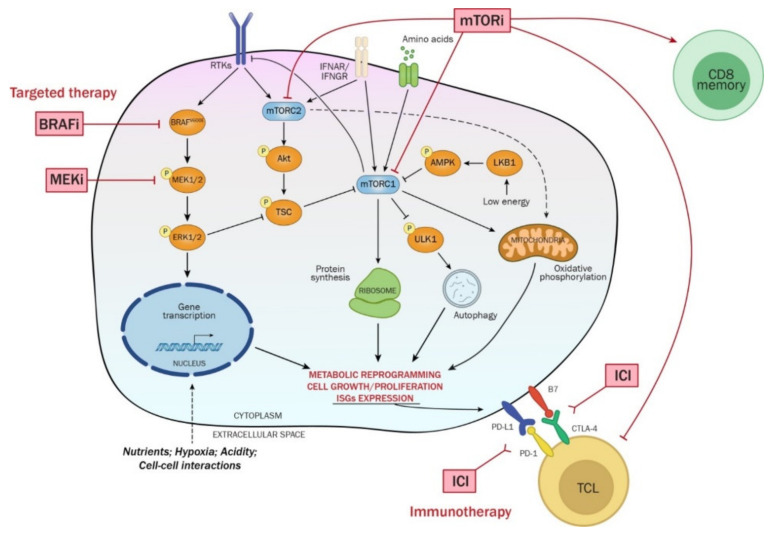
Summary of the cellular processes regulated by mTOR pathway and its connections with the BRAF/MEK/ERK signaling cascade. mTOR complexes are activated by various extra- and intracellular stimuli related to the energetic status of the cell and nutrient availability in the extracellular space. mTORC1 functions can be regulated by the BRAF pathway via an ERK-dependent mechanism and mTORC1 itself exerts a negative feedback mechanism on RTKs signaling, inhibiting mTORC2 and BRAF/MEK/ERK pathways. mTOR complexes support therapeutic resistance of melanoma cells by boosting ATP production of the mitochondria, by enhancing protein synthesis. mTORC1 and mTORC2 also promote the expression of Interferon-stimulated genes (ISGs), potentially modulating anti-tumor immune responses. Note that mTORC1 inhibition can play a role in the switch toward therapeutic resistance by removing a brake on autophagy. Additionally, systemic mTOR inhibition can play both immunosuppressive and immunostimulatory roles by impairing CD8+ cytotoxic T cells (TCL) and by favoring the expansion of CD8+ memory T cells (CD8 memory). i: inhibitors; ICI: immune checkpoint inhibitors; AMPK: AMP-activated protein Kinase; TSC: tuberous sclerosis; IFNAR/IFNGR: interferon receptors.

Activation of the RAS/BRAF/MAPK cascade, driver event in melanoma, can engage mTORC1 signaling via multiple mechanisms, including the activation of PI3K/Akt signaling downstream of RAS, and direct inactivation of the GAP activity of TSC1/TSC2 complexes towards Rheb via ERK-dependent TSC2 phosphorylation [277,278,279] (Figure 3).

A fraction of human melanomas derives from preexisting melanocytic nevi, and mutations leading to a constitutive activation of the MAPK signaling are also found in the majority of human nevi [280]. Karbowniczek et al. compared in nevi and melanomas the phosphorylation of S6 ribosomal protein (an S6K1 substrate) as readout of mTORC1 activation. They found that although the majority of nevi bear mutations that activate the RAS/MAPK axis, the vast majority of benign lesions are negative (or modestly positive) for mTORC1 activation, whereas more than 70% of melanomas display strong mTOR-dependent S6 phosphorylation [281]. Consistently, the growth of several melanoma cell lines was attenuated by rapamycin treatment in vitro. This suggests that in benign nevi the activation of mTORC1 is likely curtailed as a consequence of negative feedback mechanisms elicited by sustained activation of MAPK signaling, while interruption of this feedback via additional mutations and/or non-genetic mechanisms may coincide with malignant transformation. Of note, MAPK signaling itself is also subjected to negative feedback mechanisms that restrain its overall signaling output, including the upregulation of dual-specificity MAPK phosphatases (DUSPS) [282,283]. In this context, the RAS/BRAF/MAPK signaling would initially induce a transient cell proliferation rapidly followed by growth arrest in nevi cells, via mechanisms reminiscent of the process of oncogene-induced senescence (OIS) described in vitro. This checkpoint can be subsequently overcome by the breakdown of growth suppressive mechanisms in the transition towards malignant transformation [280]. Activation of mTOR signaling is likely to occur at this stage, since activation of PI3K/Akt has been found in human contiguous nevus-melanoma specimens [284]. The idea that mTOR activation plays essential roles in melanomagenesis is also strongly supported by work on murine melanoma models [285,286,287]. Animals with a conditional expression of BrafV600E in melanocytes develop benign melanocytic lesions reminiscent of human melanocytic nevi. A concomitant loss of PTEN activates the PI3K/Akt/mTOR signaling, overcomes OIS induced by MAPK signaling, and results in melanoma formation with 100% penetrance. By crossing mice carrying a tamoxiphene-inducible Cre allele driving the conditional expression of BRAFV600E in melanocytes with a series of conditional knockout murine models, Damsky et al. dissected the transitions from pre-arrested to post-arrested to transformed melanocytic lesions in vivo [288]. Their results support a model in which mTORC1 activation (achieved by loss of its negative regulator Lkb1) is necessary for the breakdown of the OIS barrier, but not sufficient for melanoma formation downstream of BRAFV600E. However, when Lkb1 is deleted together with the Cdkn2a (Ink4A/Arf locus), thereby eliminating a major pro-senescence barrier, then melanoma rapidly occurs. The authors found that, in addition to mTORC1, a concomitant activation of the mTORC2/Akt axis is necessary for achieving unrestricted proliferation of BRAFV600E-melanoma cells. The deletion of Cdkn2a, a mutation that frequently occurs in all known melanoma genetic subtypes, was found associated with the activation of mTORC2/Akt signaling although the precise mechanisms underlying this event where not fully elucidated. In the context of BRAFV600E mutation, the concomitant activation of both mTORC1 and mTORC2 can also be obtained by PTEN deletion. It is worth mentioning that, in human patients, most mutations of PTEN and LKB1 co-occur with BRAF activating mutations [7]. It has been suggested that activated Ras signaling resulting from mutations of NRAS or NF1 is sufficient for mTOR activation, while Braf may require additional mutations to activate mTOR. Mutations in the CDKN2A, instead, are common in all melanoma subtypes, suggesting that the breakdown of the senescence barrier is a required step in the progression of melanoma regardless of the driver mutation [289].

It has been proposed that in melanomas that do not arise from preexisting nevi, the acquisition of the same type of mutations that drive the progression from arrested nevi to melanoma may occur in a different order. In such cases, mutations that lower the senescence barrier (Cdkn2A or PTEN inactivation) would occur first, followed by mutations in the BRAF/MAPK signaling axis [280].

The involvement of mTORC2 in melanoma progression has been further suggested by the observation that the gene of its essential component Rictor is amplified in a subset of human melanomas [290]. Moreover, a critical role for Rictor/mTORC2 in melanoma progression has been indicated in the Braf/PTEN mouse melanoma model downstream of the DNA methyltransferase DNMT3B, which is frequently overexpressed in several tumors [291]. DNMT3B loss dramatically suppresses melanoma formation in the Braf/Pten murine model. Loss of DNMT3B was associated with hypomethylation of the miR-196b promoter resulting in increased miR-196b expression, which directly targets the mTORC2 component Rictor. Indeed, loss of Rictor expression prevented melanoma formation and progression in this context.

Overall, the above studies provide compelling evidence that both mTORC1 and mTORC2 synergize in the malignant transformation of melanocytes. Both mTOR complexes stimulate cancer cell metabolic functions by promoting glucose metabolism, energy production, lipid, and protein synthesis (for review see [242]), which collectively fuel the expansive growth of malignant melanoma.

### 8.3. mTOR Signaling in Therapeutic Resistance to BRAFi/MEKi

Activation of the PI3K/Akt/mTOR signaling axis has been implicated in therapeutic resistance and metabolic reprogramming of many tumor types in response to both chemotherapy and targeted therapies [279] (Figure 3). Villanueva et al. reported that BRAF-mutated melanoma cells that develop resistance to BRAFi display increased phosphorylation of IGF1-R at Tyr1131, which is indicative of kinase activation, and that simultaneous MEK and IGF-1R/PI3K inhibition leads to cytotoxicity in melanoma cells resistant to BRAFi [292]. A similar scenario was reported by Atefi et al. that identified the activation of Akt/mTOR signaling as a prominent mechanism of cross-resistance to BRAF and MEK inhibitors that can be reversed in vitro by the concomitant pharmacological inhibition of Akt or mTOR in both patient-derived and established melanoma cell lines [293]. Shi et al. in the attempt to identify early events occurring in patient’s melanomas shortly after exposure to BRAFi or BRAFi/MEKi, found that Akt activity is engaged in drug-naïve tumors shortly after the beginning of treatments [294]. Akt activation upon BRAF inhibition is most likely the result of the interruption of a tonic negative feedback fueled by constitutive BRAF signaling on RTKs, such as PDGFR, EGFR, or IGF-1R, whose expression/activity often rebounds during the onset of adaptive resistance [292,295,296]. Moreover, Chen et al. also found that in melanoma cells, the BRAFV600E protein interacts with Rictor and restrains both mTORC2 and Akt activation, suggesting the existence of multiple layers of regulation through which active BRAF attenuates the overall output of RTK/PI3K/Akt mTOR signaling in drug-naïve melanoma cells [297]. Overall, Akt activation in response to BRAFi in drug-naïve tumors likely represents an adaptive response to the treatment that fosters survival of drug-tolerant persister cell populations and that prepares the ground for additional resistance mechanisms (mutations) that hallmark the transition from adaptive to acquired resistance. Of note, some of these de novo mutations can promote constitutive activation of Akt signaling, thereby uncoupling Akt signaling from adaptive responses [294].

Since both BRAF/MAPK and PI3K/Akt signaling axes impinge on mTORC1 activity in the presence of sufficient amino acid levels [242] (Figure 3), mTORC1 activation in response to BRAF or MEK inhibition has been monitored in BRAFi sensitive and BRAFi resistant BRAFV600E melanoma cells [298]. Using as readout of mTORC1 activation the phosphorylation levels of ribosomal protein S6, the authors found that in sensitive cells and tumors, downregulation of mTORC1 rapidly occurs in response to BRAF inhibition, whereas resistant cells fail to downregulate mTORC1, and this was unrelated to the level of ERK signaling inhibition. Importantly, in paired biopsies obtained from patients with BRAF-mutant melanoma before treatment and after initiation of BRAF inhibitor therapy, the suppression of S6 phosphorylation was found to be predictive of improved progression-free survival.

Shi et al. found that in PDGFRβ-upregulated, Vemurafenib-resistant BRAF-mutated melanoma cell lines, a combined BRAF, PI3K, and mTORC1/2 inhibition suppresses the compensatory signaling at the basis of therapeutic resistance, resulting in a highly synergistic growth inhibitory response in vitro [299]. However, such drug combinations did not result in an efficient cytotoxic response. In contrast, the combination of MEK1/2, PI3K, and mTORC1/2 inhibitors was very effective in triggering apoptosis in these targeted therapy resistant cells. Interestingly, both the mTORC1/2 inhibitor AZD8055 and the dual PI3K/mTORC1/2 inhibitor BEZ235 used in combination with Vemurafenib, resulted in a faster recovery of ERK phosphorylation in treated cultures compared to Vemurafenib alone, whereas in the presence of the MEK1/2 inhibitor AZD6244, ERK activation was maintained long term and reflected the higher levels of apoptotic cell death triggered by this drug combination. The fact that PI3K and/or mTORC1/2 inhibition in the presence of Vemurafenib accelerates the recovery of ERK activation suggests that at least in some contexts, an elevated mTORC1/2 activity can prolong the duration of BRAF (but not MEK) inhibition. mTOR inhibition was also found to synergize with the combinatorial treatment with dabrafenib and trametinib in inducing growth inhibition in vitro in BRAF-mutated cell lines with acquired resistance to BRAF inhibition caused by mutations in NRAS or MEK oncogenes [300]. In a recent study, Wang et al. have monitored the levels of mTORC1 activation in a panel of paired parental and BRAFi/MEKi resistant (CR) cell line cells in response to this combination of inhibitors. Consistent with previous work on BRAFi resistant cells [298], they found that mTORC1 activity was suppressed by the combinatorial treatment in parental cells but was substantially unaffected in double-resistant cells. A similar scenario was also described in tumor xenografts in vivo, in which the therapeutic response of tumors to the combinatorial therapy was found to be related to the degree of mTORC1 inhibition. The authors also provided evidence that the combination of BRAFi/MEKi and Rapamycin induce cell cycle arrest and apoptosis in CR cell lines and potently suppress the growth of xenografts generated by CR cells in vivo. Moreover, the mechanism of activation of mTORC1 in CR cell was found dependent on the genetic PTEN status of CR cells. In PTEN-/- cells, mTORC1 was found exquisitely dependent on upstream Akt activation, in PTEN +/- cells both Akt and ERK contribute to mTORC1 activation while in PTEN+/+ cells ERK activation plays a predominant role in mTORC1 activation, which was consistent with the differential sensitivity of cells to ERK or Akt inhibition.

### 8.4. mTOR Activation and the OXPHOS Switch in BRAFi/MEKi Resistant Melanoma

As previously described, the shift between glycolytic-based to OXPHOS mitochondrial energetic metabolism is a frequent feature in a substantial proportion of targeted-therapy resistant melanomas. Upregulation of the MITF/PGC-1α in melanomas promotes mitochondrial energy metabolism and a concomitant increase in ROS detoxification capacity, that enable cancer cells to withstand oxidative damage and to survive under stressful conditions [27]. Gopal et al. found that a subset of BRAF or NRAS mutated melanomas and cell lines resistant to BRAF and/or MEK inhibition that displayed activation of the Akt pathway also exhibited an increased an OXPHOS transcriptional signature [301]. Knockdown of PGC-1α, the master transcriptional regulator of nuclear encoded electron transport chain gene transcription, was sufficient to revert the resistant phenotype of cells. All MEK-inhibitor resistant cell lines in this study could be re-sensitized to MEK inhibition by a concomitant pharmacological or genetic mTORC1/2 inhibition. Thus, in this context, mTORC1 and mTORC2 apparently synergize in promoting the PGC-1α-dependent OXPHOS switch (Figure 3). Mechanistically, the authors found that in both BRAF and NRAS mutated melanomas, MEK inhibition increases MITF expression, which, in turn, upregulates the expression of PGC-1α. mTORC1/2 inhibition caused a cytoplasmic sequestration of MITF, a decreased PGC-1α expression and reduced OXPHOS. Therefore, considering the prominent role of mitochondria in the onset of melanoma therapeutic resistance [302], mTOR signaling seems to be involved in the regulation of this process at the switch between sensitivity and resistance to BRAFi/MEKi. However, since both mTORC1 and mTORC2 can be involved, both in the early phases of melanocyte transformation (frequently associated with an aerobic glycolysis-based bioenergetics coupled with BRAFi sensitivity) and in the subsequent onset of BRAFi/MEKi resistance (predominantly characterized by an OXPHOS bioenergetic modality), the roles of mTORC1 and mTORC2 may be complex and highly context-dependent. In fact, mTORC1 activity can stimulate glycolysis (for review see [242]), but also promote mitochondrial metabolism by enhancing the translation of nuclear-encoded Electron Transport Chain components [303]. Moreover, mTORC1 positively regulate the glutaminolytic anaplerotic pathway, which fuels TCA cycle and mitochondrial OXPHOS, and this pathway is preferentially engaged in targeted therapy-resistant melanomas [149]. Glutaminolysis, in turn, can also activate mTORC1 signaling, potentially creating a feed-forward loop that maintains elevated mTORC1 activity [304].

Transformed cells that are addicted to mTORC2 signaling have been found to depend on mitochondrial functions [305]. On the other hand, mTORC2 is a positive regulator of glycolysis and lipid biogenesis via both Akt dependent and independent mechanisms (for review see [306]). However, in different cell types and tissues, mTORC2 deficiency induced by Rictor conditional ablation was found associated with an OXPHOS gene expression signature [307], with increased mitochondrial functions [303] and with increased glutamine consumption through the glutaminolytic pathway [308,309]. Thus, it seems likely that, in melanoma, changes in the overall scenario induced by therapeutic regimens, modifications in the tumor microenvironment or acquisition of additional genetic alterations likely direct mTORC1 and mTORC2 on different subsets of downstream targets and cellular bioenergetics processes during the progression of BRAF-mutated melanoma towards therapeutic resistance. This picture is further complicated by the coexistence within the same tumor of cell populations with different bioenergetic needs and metabolic profiles. Very recent work suggests that post-transcriptional regulation of metabolic genes following BRAF inhibition plays a central role in the adaptation of BRAFV600E melanoma cells via translational reprogramming [310]. Given the central role of mTORC1 signaling in the regulation of mRNA translation, it is likely that this signaling complex may be involved in this newly discovered adaptive mechanism of resistance to targeted therapy.

### 8.5. mTORC1/2 Activation or Inhibition and Immune Therapies in Melanoma

As previously stated, IFNs play essential roles in the resistance/response of melanoma patients to ICI [224] and both mTORC1 and -2 are key players in the modulation of IFN signaling [311,312]. The identification of biomarkers of sensitivity/resistance of melanoma patients to ICI is regarded as an urgent medical need. Considering the overall implications of mTOR signaling in mediating IFN biological effects, it will be important to determine whether, in treatment-naïve melanoma patients, a high- or low intratumoral activity of mTORC1 or mTORC2 could help predict the therapeutic response to ICI. Should this be the case, the analysis of the phosphorylation levels of mTORC1 and -2 protein targets in patients’ biopsies, along with the definition of the immune cell infiltrate would represent a feasible methodology for orienting the choice of the most appropriate first-line treatment between ICI and targeted therapy in patients bearing BRAF-mutated melanomas.

In addition, modulation of mTOR signaling can also alter the response to ICIs. Despite the overall immunosuppressive role of Rapamycin, mTOR inhibition with Rapalogs has been shown to favor the expansion of CD8+ memory T cells [313] and to have a synergistic effect with different types of immunotherapies in some preclinical tumor models. In particular, a potentiating effect of Rapamycin has been reported in the context of cancer vaccination using the transplantable B16 murine melanoma model [314,315,316]. The possibility to combine immune therapies and mTOR pharmacological inhibitors represents an exciting new venue in research that has extensively been reviewed elsewhere [254,317]. However, data on the effects of combinations of mTOR inhibitors with ICI on melanoma models are currently missing. Considering the multifaceted effects of mTOR inhibitors on different immune cells populations, a safe use of these drugs in combination with currently available immunotherapies for melanoma (ICI) will likely require extensive validation and schedule optimization in both preclinical and clinical settings, and needs also to take into account individual differences in the cellular composition of the immune cell tumor infiltrate.

## 9. The Crosstalk between NAD Metabolism and mTOR Signaling

Since both the activation of mTOR and NAD metabolism via NAMPT are involved in the therapeutic resistance of melanoma to BRAFi/MEKi, an outstanding question is whether in this context these two these two key molecular pathways are functionally interconnected. Although experimental data in melanoma to our knowledge are currently missing, in hepatocarcinoma, pancreatic cancer, and multiple myeloma models pharmacological inhibition of NAMPT results in an inhibition of mTORC1 activity [213,318,319]. Mechanistically, the collapse of ATP synthesis caused by NAMPT inhibition activates AMPK, which, in turn, restrains mTORC1 activity. Of note, in these experimental settings, NAMPT and mTOR inhibition synergized in inducing cytotoxicity in cancer cells. Another potential interconnection between NAD metabolism and mTOR signaling is at the level of the mTORC2 target Akt that has been recently demonstrated to phosphorylate and activate NADK, the enzyme that converts NAD into NADP. NADP, in turn, is essential for producing NADPH, the primary cofactor for reductive metabolism [320]. However, future work is needed to verify whether a combinatorial treatment based on concomitant NAMPT and mTOR inhibition represents a feasible strategy for targeting BRAFi/MEKi-resistant melanoma.

## 10. Conclusions and Perspective

In recent years, the development of targeted small-molecule inhibitors and immunotherapy has revolutionized the care and improved the overall survival of melanoma patients. Despite these landmark changes in practice, the majority of patients are either intrinsically resistant or rapidly acquire resistance to MAPK pathway inhibitors and immune checkpoint blockade. Common resistance mechanisms overcome the dependence of tumor cells on initial BRAF oncogenic signaling during targeted therapy, and permit evasion of the host immune system to allow melanoma growth and survival following immune checkpoint blockade. This suggests: (i) the limitation in the long-term use of current therapeutic strategies to treat melanoma patients due to the rapid onset of resistant disease; and (ii) the necessary requirement for personalized treatment regimens that take into account patient-intrinsic genetic and immunologic characteristics. Here, we summarized how NAD/NAMPT and mTOR signaling are typically involved in melanoma progression and therapeutic resistance to BRAFi/MEKi, but also could be implicated at different levels in the complex interplay between different immune cell populations that can potentially impact on the patients’ response to ICIs, even if experimental data on this particular aspect are currently scarce in the context of melanoma. Additionally, the functional and molecular connections between NAD/NAMPT-dependent metabolic/epigenetic reshuffling and mTOR signaling that have been indicated in tumor models other than melanoma is an open venue of future investigation. The possibility to inhibit these two players in combination with BRAFi/MEKi and/or ICIs to contrast/overcome drug resistance is appealing and deserves experimental investigation. However, it is likely that simultaneous “vertical” inhibition of pleiotropic signaling/metabolic pathways, such as those downstream of MAPK, mTOR, and NAMPT, will be challenging in clinical settings, as they could result in toxic effects that cannot be predicted based solely on pre-clinical models. Nevertheless, the lesson that we learned on cancer therapeutic resistance tells that tumors change continuously over time in response to therapy. Thus, we probably need to modify our therapeutic strategies taking into account these temporal changes in the metabolic and immunological landscape of tumors. The elucidation of both the determinants and timing of activation of melanoma resistance mechanisms may allow a rational design of a sequential use of MAPKi, NAMPTi, mTORi, and/or ICI rather than their bare simultaneous administration. Moreover, the identification of the molecular effector mechanisms acting downstream of both the NAD/NAMPT and mTOR pathways will likely pave the way for the development of less toxic, more specific strategies to tackle melanoma therapeutic resistance.

## Data Availability

Not applicable.
